# Pan‐cancer analysis identifies *CD300* molecules as potential immune regulators and promising therapeutic targets in acute myeloid leukemia

**DOI:** 10.1002/cam4.4905

**Published:** 2022-05-31

**Authors:** Zi‐jun Xu, Ye Jin, Xin‐long Zhang, Pei‐hui Xia, Xiang‐mei Wen, Ji‐chun Ma, Jiang Lin, Jun Qian

**Affiliations:** ^1^ Laboratory Center Affiliated People's Hospital of Jiangsu University Zhenjiang Jiangsu People's Republic of China; ^2^ Zhenjiang Clinical Research Center of Hematology Zhenjiang Jiangsu People's Republic of China; ^3^ The Key Lab of Precision Diagnosis and Treatment in Hematologic Malignancies of Zhenjiang City Zhenjiang Jiangsu People's Republic of China; ^4^ Department of Hematology Affiliated People's Hospital of Jiangsu University Zhenjiang Jiangsu People's Republic of China; ^5^ Department of Hematology The People's Hospital of Danyang, Affiliated Danyang Hospital of Nantong University Danyang Jiangsu People's Republic of China

**Keywords:** *CD300s*, immune evasion, leukemia, pan‐cancer, prognosis

## Abstract

**Background:**

*CD300s* are a group of proteins playing vital roles in immune responses. However, much is yet to be elucidated regarding the expression patterns and clinical significances of *CD300s* in cancers.

**Methods:**

In this study, we comprehensively investigated *CD300s* in a pan‐cancer manner using multi‐omic data from The Cancer Genome Atlas. We also studied the relationship between *CD300s* and the immune landscape of AML.

**Results:**

We found that *CD300A*‐*CD300LF* were generally overexpressed in tumors (especially AML), whereas *CD300LG* was more often downregulated. In AML, transactivation of *CD300A* was not mediated by genetic alterations but by histone modification. Survival analyses revealed that high *CD300A*‐*CD300LF* expression predicted poor outcome in AML patients; the prognostic value of *CD300A* was validated in seven independent datasets and a meta dataset including 1115 AML patients. Furthermore, we demonstrated that *CD300A* expression could add prognostic value in refining existing risk models in AML. Importantly, *CD300A*‐*CD300LF* expression was closely associated with T‐cell dysfunction score and could predict response to AML immunotherapy. Also, *CD300A* was found to be positively associated with *HLA* genes and critical immune checkpoints in AML, such as *VISTA*, *CD86*, *CD200R1*, *Tim‐3*, and the *LILRB* family genes.

**Conclusions:**

Our study demonstrated *CD300s* as potential prognostic biomarker and an ideal immunotherapy target in AML, which warrants future functional and clinical studies.

## Graphical abstract

We found that *CD300A*‐*CD300LF* were significantly upregulated and high expression of these genes predict worse survival in AML. Specifically, *CD300A* may aid risk stratification in AML and its expression was likely to be regulated by H3K4me3 histone modification. Importantly, we demonstrated *CD300A* as an essential co‐inhibitory signal that might cause NK cell exhaustion and promotes immune escape of AML cells.

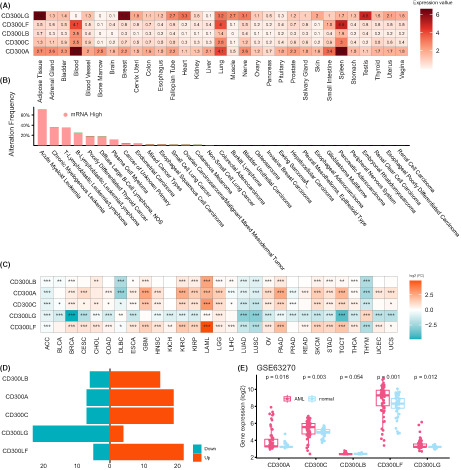

## 1 INTRODUCTION

Acute myeloid leukemia (AML) is a heterogeneous disease with underlying cytogenetic or molecular genetic aberrations. Despite progress in our understanding of the biology of this disease, chemotherapy remains as the main intervention and most patients will inevitably relapse and ultimately die. Therefore, there is an urgent need to uncover more effective therapeutic targets.

Targeting immune checkpoints has proved to be beneficial in treating various cancers and inhibitors blocking *PD‐1*/*PD‐L1* have already been approved by FDA.^1^, ^2^, ^3^ AML was by itself poorly immunogenic and extremely immune‐suppressive.^4^ This could be caused by a number of tumor immune evasion mechanisms, including upregulation of co‐inhibitory ligands such as *CTLA‐4*, *PD‐L1*, *PD‐1*, *Tim‐3* on AML cells,^5^ reduced expression of neoantigens/MHC,^6^ enrichment of immunosuppressive cell subsets such as regulatory T cells (Tregs),^7^ myeloid‐derived suppressor cells (MDSCs)^8^, ^9^, and tumor‐associated macrophages (TAMs),^10^, ^11^and induction of T‐cell exhaustion.^5^, ^12^ Several clinical and preclinical studies have demonstrated the promise of blocking co‐inhibitory receptors in AML, yet the efficacy and response rate remain to be completely determined.^5^ Indeed, these studies have paved the way for rigorous study of co‐inhibitory molecules in AML.

The human *CD300* receptor family composed of seven members (*CD300A*, *CD300LB*, *CD300C*, *CD300LD*, *CD300E*, *CD300LF*, and *CD300LG*) located on chromosome 17.^13^, ^14^ Specifically, the cytoplasmic domains of two receptors, *CD300A* and *CD300LF*, contain the immunoreceptor tyrosine‐based inhibitory motifs (ITIMs), which endows them with inhibitory‐dominant potential in immune processes.^15^ The *CD300* molecules, especially *CD300A* and *CD300LF*, are mainly expressed on myeloid cells, such as monocytes, macrophages, and dendritic cells (DCs).^13^, ^15^, ^16^ In particular, *CD300A* is also expressed on the tumor‐suppressive NK and CD8+ T cells, in which it mediates inhibitory signal and leads to an exhaustion status of these cell.^17^, ^18^, ^19^, ^20^

It has been reported that *CD300* family members is involved in multiple autoimmune disorders, such as asthma,^21^ colitis,^22^, ^23^ acute kidney injury,^24^ and brain damage.^25^ However, far too little attention has been paid to the relation between *CD300s* and cancers, which are also immune‐mediated or inflammatory diseases. There are only few evidences that *CD300A* was found to be significantly overexpressed in hematological malignancies, such as acute lymphoblastic leukemia (ALL),^26^, ^27^ AML,^28^ and diffuse large B‐cell lymphoma (DLBCL).^29^ Therefore, in this study, we performed a systematic analysis concerning the expression patterns, transcriptional regulations, clinical impacts, and roles in the tumor microenvironment (TME) of *CD300* members in a broad spectrum of cancer types, focusing on its role in AML.

## 2 MATERIALS AND METHODS

### 2.1 Analysis of gene expression data

The transcript levels of *CD300* family in normal tissues were determined by using the Genotype‐Tissue Expression (GTEx) dataset. Averaged expression data of *CD300s* for over 1000 cancer cell lines from various organ sites were analyzed through cBioPortal (https://www.cbioportal.org/).We then systematically analyzed the expression patterns of *CD300s* between 9197 tumor and 8290 normal samples by combining RNA sequencing data from the TCGA and the GTEx projects. The two datasets were downloaded from the UCSC Xena project and were normalized between arrays using the limma package.^30^ The UALCAN (http://UALCAN.path.uab.edu/) web tool was used to evaluate protein expressions of *CD300s* in certain cancers and adjacent normal tissues. Further, we retrieved a dataset containing both healthy and AML samples from Gene Expression Omnibus (GEO) (https://www.ncbi.nlm.nih.gov/geo/) (accession number GSE63270) to validate the differential expression of *CD300s* between AML and normal controls. We also used the Hemap AML dataset to analyze whether *CD300s* expressions were associated with certain molecular subtypes.^31^ The Human Proteome Map (https://www.humanproteomemap.org/) database was used to assess protein expression levels of *CD300s* in normal tissues and cell types, including 17 adult tissues, six primary hematopoietic cells, and seven fetal tissues.

### 2.2 Analysis of AML single‐cell RNA‐sequencing (scRNA‐seq) data

To quantify the expression of *CD300s* across immune cells at the single‐cell level, we utilized two published scRNA‐seq data: one consists of 30,712 bone marrow (BM) cells from 16 AML samples at diagnosis (Van Galen AML scRNA, GSE116256),^32^ and the other consists of 30,579 AML BM cells for eight patients (FIMM AML scRNA). Both datasets were obtained through the Synapse Web Portal (https://www.synapse.org and doi: 10.7303/syn21991014), and were processed and visualized using custom scripts provided by Dufva et al.^31^

### 2.3 Analysis of genetic and epigenetic alteration data

Genetic alteration (including somatic mutations, amplification, and deep deletion) frequencies of *CD300s* across TCGA pan‐cancers (including 10,967 patients) were analyzed and visualized through the cbioportal genomic database (http://www.cbioportal.org). Mutation data of 24 most frequently mutated genes identified in the TCGA AML project were used to determine the association between *CD300s* expression and common gene mutations. The relationships between mutation status and the dichotomized expression of *CD300s* were analyzed by two‐sided Fisher exact tests.

*CD300s* promoter methylation data in various tumor and normal samples were analyzed through DiseaseMeth database (http://bio‐bigdata.hrbmu.edu.cn/diseasemeth/analyze.html). Correlation between *CD300s* expression and methylation across pan‐cancers were assessed through the GSCALite platform (http://bioinfo.life.hust.edu.cn/web/GSCALite/).^33^

Previously published H3K4me3 ChIP‐seq data of three leukemia cells were downloaded from GEO (K562 cells from GSE74359; MLL‐AF9 blast cells from GSE89336; and KG‐1 cells from GSE109619) and visualized via UCSC genome browser.

### 2.4 Survival analysis

Univariate Cox regression was performed to examine influence of *CD300s* expression on overall survival (OS) across 33 cancers. We then used the Kaplan–Meier method to estimate survival in AML patients with high and low *CD300s* levels. The optimal cut point of *CD300s* expression was determined by the X‐tile method.^34^ The prognostic value of *CD300A* in AML was further validated in seven independent cohorts of AML patients (GSE6891, *n* = 293; GSE10358, *n* = 304; GSE37642 [U133A], *n* = 422; GSE37642 [U133plus2], *n* = 140; GSE12417 [U133A], *n* = 163; GSE12417 [U133plus2], *n* = 79; GSE71014, *n* = 104). We also combined these microarray datasets using the combat function from the sva R package to create a dataset with maximum number of samples. To examine the predictive power of *CD300A* expression in the context of existing models, we recalculated two gene expression‐based prognostic models (LSC17 and LI24) as previously described.^11^ The survivalROC R package was used to estimate the time‐dependent receiver operating characteristic (ROC) curve from survival data and compute the value of the area under the curve (AUC). A multivariable model was used to develop a nomogram in the TCGA cohort, and the scores of each variable were calculated and visualized using the nomogramEx R package.

### 2.5 Immune response analysis

Immune cell type abundances were estimated with CIBERSORT as described earlier.^11^ We also used other algorithms^35^ available from TIMER 2.0 web portal (http://timer.comp‐genomics.org/) to quantify the proportions of monocytes, macrophages, and CD8 T cells. Finally, the TIDE (Tumor Immune Dysfunction and Exclusion) database (http://tide.dfci.harvard.edu) was used to calculate T‐cell dysfunction, T‐cell exclusion, and specific immune signature scores, and to predict the potential response to immunotherapy in AML.

### 2.6 Differential gene expression analysis and functional enrichment analysis

Briefly, differentially expressed genes between high and low *CD300A* expressers were defined using DESeq2 R package at false discovery rate (FDR) <0.05. Gene Ontology (GO) analysis, Kyoto Encyclopedia of Genes and Genomes (KEGG), and Reactome pathway analysis of the differentially expressed genes (DEGs) were performed using the STRING database (http://www.string‐db.org/). GO, KEGG, and Reactome terms with false discovery rate (FDR)‐corrected p‐values less than 0.05 were considered as significantly enriched. The web‐tool STRING (http://string.embl.de/) was used to construct a protein–protein interaction (PPI) network of the DEGs. A confidence score >0.9 was used as the judgment criterion. Results were displayed with Cytoscape v3.8.2. We also used GeneMANIA (http://genemania.org/) to search for gene interactions between *CD300* members and co‐expressed genes. Gene set enrichment analysis (GSEA) was performed via GSEA v4.1.0 software (http://www.broad.mit.edu/gsea) using the Hallmark gene sets within the Molecular Signatures Database (MSigDB).

### 2.7 Statistical analysis and visualization

Wilcoxon rank sum tests were used to compare differences between two groups. Specifically, differential expression of *CD300s* between each molecular subtype and the remaining samples in the Hemap dataset were analyzed as previously described.^31^ Spearman correlation analysis was used to test the association of *CD300s* with HLA genes^36^ and immune checkpoints^37^ in AML. All statistical analyses and visualizations were performed using R statistical software, version 4.1.1. The box, violin, bar, and bubble plots were generated with the R package “ggplot2”, “ggpubr”, and “ggsci”, the volcano plot was created using the “EnhancedVolcano” package, and survival curves were plotted using the “survival” package. All statistical tests were two‐sided with p‐values less than 0.05 considered significant.

## 3 RESULTS

### 3.1 Expression patterns of CD300s in normal tissues and cancer cell lines

**FIGURE 1:**
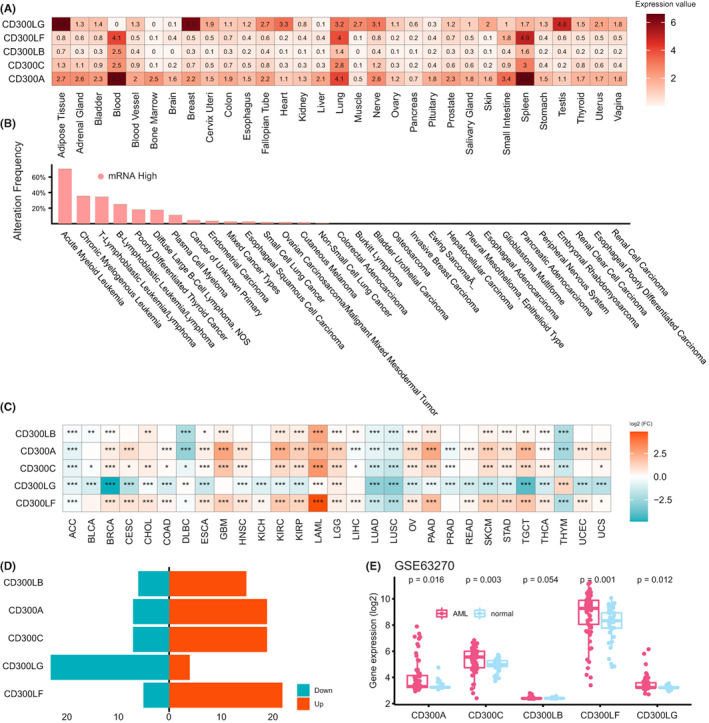
Expression patterns of *CD300s* in normal tissues, cancer cell lines, and primary tumor samples. (A) Heatmap showing mRNA expression levels of *CD300s* in normal tissues from the Genotype‐Tissue Expression (GTEx) database. (B) Bar plot showing transcriptional alteration frequencies of *CD300s* in various tumor cell lines from the Cancer Cell Line Encyclopedia (CCLE) database. (C) Heatmap of differential expression profiles of *CD300s* between tumor and normal samples, combining data from TCGA and GTEx databases. The color depicts the log2‐transformed fold change (Log2FC) between tumor and normal tissues. *p < 0.05; **p < 0.01; ***p < 0.001. (D) Bar plot showing genes significantly upregulated and downregulated (*P* < 0.05) across different cancer types. Red, upregulated expression; blue, downregulated expression. (E) Box plots showing expression levels of *CD300s* in normal controls and AML in the GSE63270 dataset

Among seven *CD300* molecules (*CD300A*, *CD300LB*, *CD300C*, *CD300LD*, and *CD300E*, *CD300LF*, and *CD300LG*). We selected only five members (*CD300A*, *CD300LB*, *CD300C*, *CD300LF*, and *CD300LG*) for our analyses. Since the probes of *CD300LD* and *CD300E* were not found in the GEO microarray data sets. First, we explored the expression patterns of *CD300s* in different human tissues based on RPKM values using GTEx (http://www.GTExportal.org/home/). We observed that *CD300A*‐*CD300LF* were highly expressed in blood, lung, and spleen; while *CD300A* was expressed broadly on most tissues, *CD300B*, *CD300C*, and *CD300LF* were only weakly expressed in other tissues (Figure 1A). Interestingly, *CD300LG* showed a unique expression pattern: it was expressed at high levels in the adipose tissue, breast, heart, and testis, but it was not expressed by blood. Next, we analyzed the expression profiles of *CD300s* in cancer cell lines from Cancer Cell Line Encyclopedia (CCLE). As shown in Figure 1B, *CD300s* showed relatively high expression in cell lines of malignant hematological cell lines (AML, chronic myelogenous leukemia, T‐lymphoblastic leukemia/lymphoma, and B‐lymphoblastic leukemia/lymphoma), and the highest expression was seen in AML.

### 3.2 Analysis of CD300 family gene expression levels in tumor and non‐tumor tissues

To date, a comprehensive pan‐cancer analyses determining the dysregulations of *CD300s* between tumor and normal tissues is still lacking. Here, we combined data from TCGA and GTEx to systematically compare *CD300s* expression between tumor and adjacent normal tissue across 29 cancer types (9197 tumor and 8290 normal samples). Surprisingly, we identified significant differential expression of *CD300s* in almost all cancer types tested (Figure 1C). Overall, *CD300A*‐*CD300LF* were more often upregulated in cancers; whereas downregulation of *CD300LG* in tumors was more commonly seen (Figure 1D). For *CD300A*‐*CD300LF*, the most remarkable difference was observed between AML and normal its normal counterparts (Figure 1C). This difference was also validated in an independent microarray dataset (GSE63270; AML, *n* = 62, Normal, *n* = 42) (Figure 1E).

In addition, we found that *CD300A*‐*CD300LF* were highly expressed in glioblastoma multiforme (GBM), brain lower grade glioma (LGG), kidney renal clear cell carcinoma (KIRC), kidney renal papillary cell carcinoma (KIRP), ovarian cancer (OV), pancreatic adenocarcinoma (PAAD), skin cutaneous melanoma (SKCM), stomach adenocarcinoma (STAD), and testicular germ cell tumor (TGCT), whereas they were markedly decreased in lung adenocarcinoma (LUAD), lung squamous cell carcinoma (LUSC), and thymoma (THYM), as compared with normal controls (Figure 1C). Further, we used the UALCAN (http://UALCAN.path.uab.edu/) database to assess the protein expression of *CD300A* in a number of solid cancers, such as breast cancer (BRCA), GBM, LUAD, KIRC, and ovarian cancer (OV) (except for *CD300LF* in GBM, other *CD300* members were not identified in these proteomic datasets). Importantly, we were able to confirm the upregulation of *CD300A* in BRCA, GBM, head and neck squamous cell carcinoma (HNSC), KIRC, PAAD, uterine corpus endometrial carcinoma (UCEC), and the downregulation of *CD300A* in liver hepatocellular carcinoma (LIHC) (except for LUAD and OV, which showed an opposite pattern of protein expression) (Figure 1C and Figure S1). Moreover, *CD300LF* showed increased expression pattern in the proteomic dataset of GBM similar to what has been observed in the RNA‐seq dataset (Figure S1). Overall, these results demonstrated that the protein expression patterns of *CD300A* in most cancers agreed very well with the observed levels of mRNA.

### 3.3 The genetic and epigenetic features of CD300s in pan‐cancers

**FIGURE 2:**
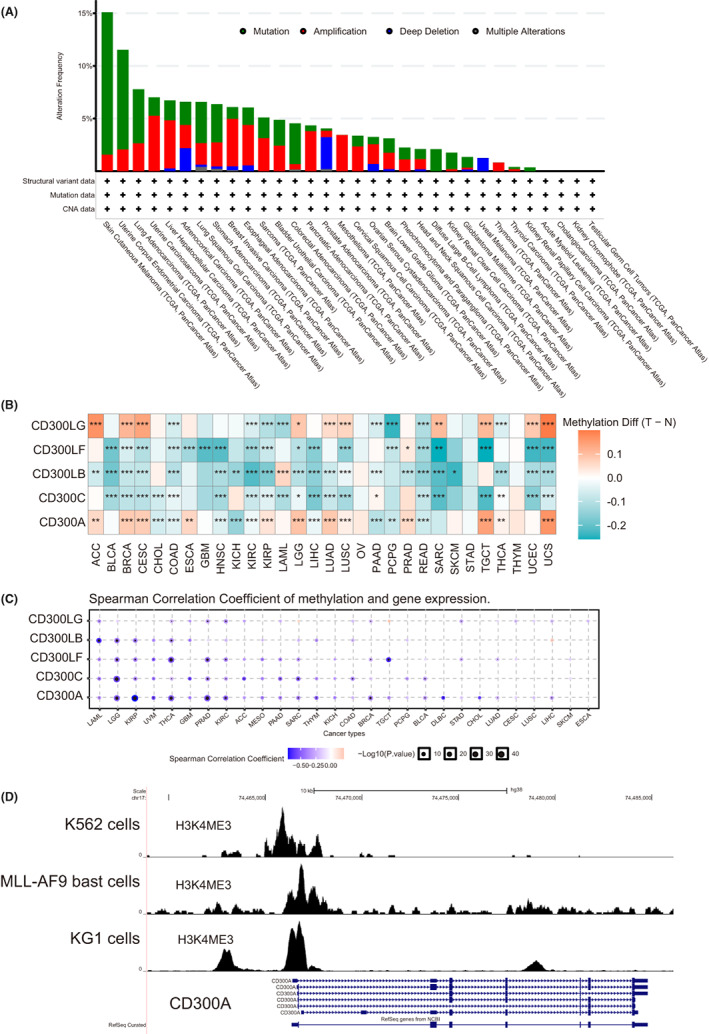
The genetic and epigenetic features of *CD300s* in pan‐cancers. (A) Genetic alteration ((including mutations, amplifications, and deletions) frequencies of *CD300s* across different tumors from TCGA. (B) Heatmap of differential methylation profiles of *CD300s* between tumor and normal samples, using data from the Diseasemeth database. The color depicts methylation differences between tumor (T) and normal (N) tissues. *p < 0.05; **p < 0.01; ***p < 0.001. (C) Correlation between methylation and mRNA expression of *CD300s* analyzed via the GSCALite platform (http://bioinfo.life.hust.edu.cn/web/GSCALite/). Blue dots indicate negative correlation and red indicate positive correlation. The size of the point represents the statistical significance. (D) ChIP‐seq tracks for H3K4me3 at *CD300A* gene loci in K562 cells, MLL‐AF9 blast cells, and KG‐1 cells. ChIP‐seq data were obtained from GSE74359, GSE89336, and GSE109619

We then explored genetic alterations (including mutations, amplifications, and deletions) frequencies of *CD300s* in TCGA pan‐cancer datasets. The average alteration frequencies of five genes are summarized in Figure 2A. Mutations of *CD300s* were mainly distributed in SKCM, UCEC, and LUAD (Figure 2A). For copy number alterations (CNAs), *CD300s* were much more frequently amplificated than deleted. In mesothelioma (MESO) and THYM, amplifications were the only genetic events, while in uveal melanoma (UVM), *CD300s* were often deleted (Figure 2A). It is worth noting that in AML, where *CD300s* were transcriptionally active, no genetic alterations were observed, suggesting other mechanisms might contribute to the abnormal *CD300s* expression in AML.

We next asked whether DNA methylation regulates the expression of *CD300s* in cancers. To this end, we obtained curated DNA methylation microarray data of *CD300s* across 30 cancer types with matched controls through the human disease methylation database Diseasemeth version 2.0 (http://bio‐bigdata.hrbmu.edu.cn/diseasemeth/). We found that *CD300LB*, *CD300C*, *and CD300LF* were significantly hypomethylated in almost all cancer types as compared to normal samples (Figure 2B). Interestingly, *CD300A*, which is upregulated in most cancers, demonstrated an overall hypermethylation pattern.

Further analysis using the GSCA database revealed weak negative correlation between methylation and expression levels of *CD300s* in a number of cancer types (Figure 2C). In AML, however, no methylation differences were observed for two most well‐characterized *CD300* members‐*CD300A* and *CD300LF* (Figure 2B).

It has been reported that *CD300A* could be regulated by transcription activation‐associated histone marks, such as histone H3 lysine 4 mono‐ and tri‐methylation (H3K4me1 and me3, respectively).^38^ Consistently, we found a significant enrichment of H3K4me3 marks in the promoter regions of *CD300A* gene in three types of leukemia cells from published ChIP‐seq datasets (K562 cells from GSE74359; MLL‐AF9 blast cells from GSE89336; KG‐1 cells from GSE109619) (Figure 2D). Overall, these results suggest that, at least in leukemia cells, *CD300A* expression might be regulated by histone modification.

### 3.4 Prognostic significances of CD300s in different cancers

**FIGURE 3:**
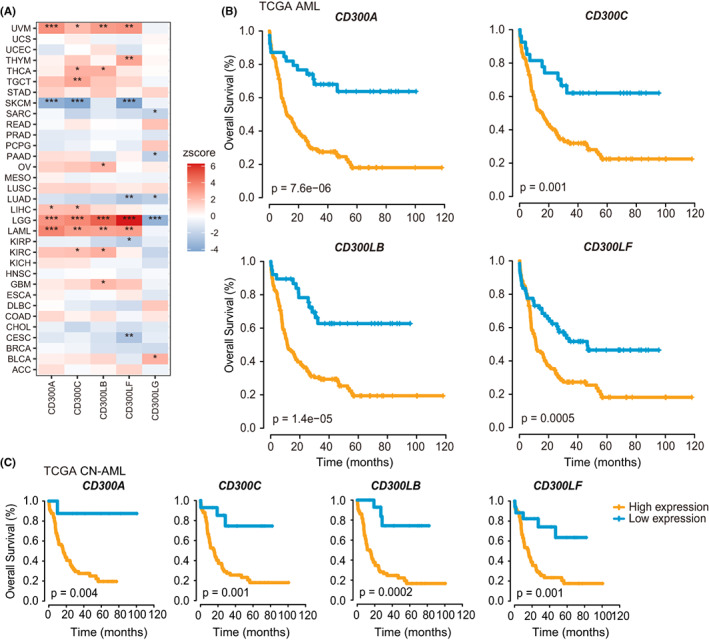
The prognostic significances of *CD300s* in cancers. (A) Association between *CD300s* expression and patient prognosis across 33 cancer types as determined by the Cox regression model. (B and C) Kaplan–Meier curves representing OS of AML patients from the whole TCGA cohort (B) and the CN‐AML subsets (C) based on the expression of indicated *CD300* members (*CD300A*‐*CD300LF*)

**FIGURE 4:**
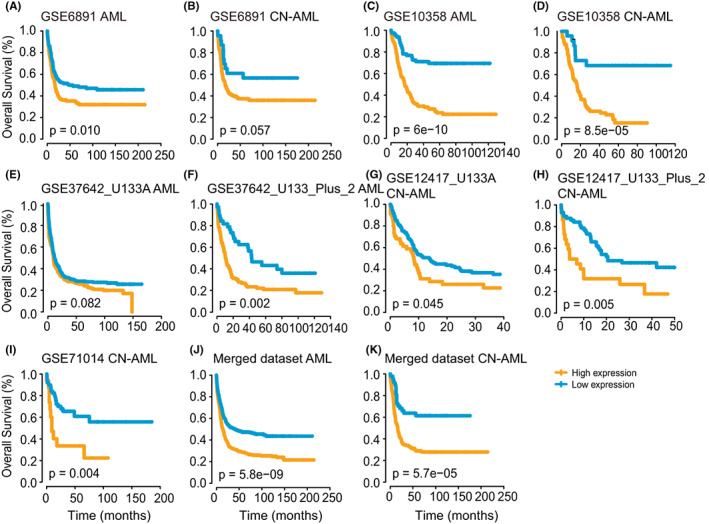
Independent validation of the prognostic value of *CD300A*. (A‐K) Kaplan–Meier curves representing OS of seven AML cohorts from GEO (GSE6891, *n* = 293; GSE10358, *n* = 304; GSE37642 [U133A], *n* = 422; GSE37642 [U133plus2], *n* = 140; GSE12417 [U133A], *n* = 163; GSE12417 [U133plus2], *n* = 79; GSE71014, *n* = 104) (A, C, and E‐I), CN‐AML patients from GSE6891 and GSE10358 cohorts (B and D), a merged dataset of 1115 AML patients (J), and 242 CN‐AML patients (K) according to *CD300A* expression status

Given that the expression of *CD300s* was significantly dysregulated in cancers, we asked whether these genes have prognostic relevance in TCGA pan‐cancer datasets. Cox regression analyses revealed that in most cancers, high expressions of *CD300A*‐*CD300LF* were related to poor overall survival (OS), such as in LAML, LGG, and UVM (Figure 3A). While in SKCM, a significant beneficial effect on OS was observed for *CD300A*, *CD300C*, and *CD300LF* (Figure 3A). However, *CD300LG* was only prognostically relevant in a few cancers (Figure 3A). Considering that *CD300A‐CD300LF* were specifically and highly expressed in AML, we decided to focus our survival analysis on these genes in AML. To this end, we collected seven independent AML datasets from GEO; X‐tile was then performed to determine the optimal thresholds in TCGA and GEO datasets. First, we were able to validate the prognostic value of *CD300A*‐*CD300LF* expression within the TCGA cohorts (Figure 3B) and cytogenetically normal (CN) subsets of AML (Figure 3C). Importantly, the adverse prognostic impact of *CD300A* was validated in seven independent cohorts of AML patients (GSE6891, *n* = 293; GSE10358, *n* = 304; GSE37642 [U133A], *n* = 422; GSE37642 [U133plus2], *n* = 140; GSE12417 [U133A], *n* = 163; GSE12417 [U133plus2], *n* = 79; GSE71014, *n* = 104) (Figure 4A,C,E‐I) and CN‐AML patients from GSE6891 and GSE10358 cohorts (Figure 4B,D). In order to incorporate the maximum number of samples, we combined these datasets from GEO yielding a meta dataset of 1115 AML patients (including 242 CN‐AML patients). In the meta dataset, *CD300A* status remained excellent predictive power for OS (whole cohort, *p* = 5.8*e^−9^; CN‐AML, *p* = 5.7*e^−5^) (Figure 4J,K).

### 3.5 Additional value of CD300A expression in refining risk stratification in AML

**FIGURE 5:**
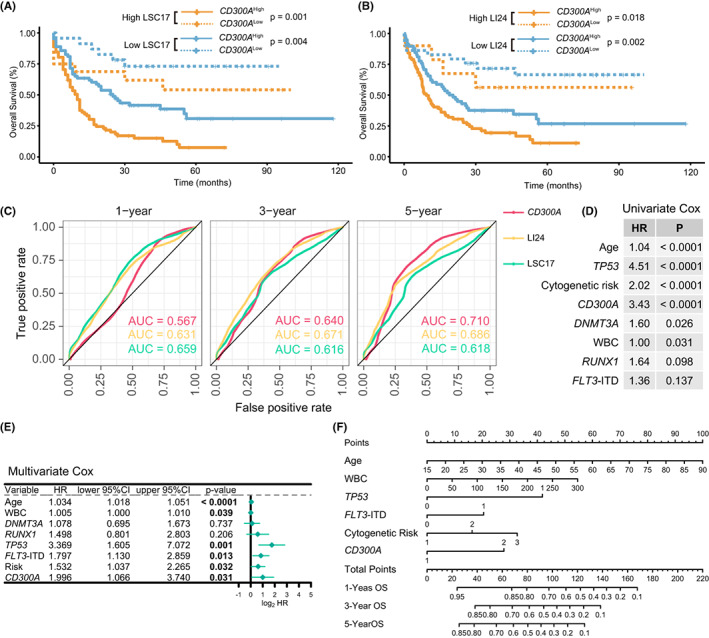
Additional value of *CD300A* expression in refining risk stratification in AML. (A and B) OS of patients from TCGA as stratified by the LSC17 (A) and the LI24 (B) signature. Patients with a low‐ and high‐risk score were further dichotomized according to *CD300A* expression status. (C) Time dependent ROC curves of *CD300A* expression, LSC17, and LI24 in the TCGA cohort at 1, 3, and 5 years. (D and E) Univariate (D) and multivariate (E) analysis of *CD300A* expression for OS in the TCGA cohort. For univariate analysis, only variables with *p* ≤ 0.20 are shown. Please see Supplementary Tables S1 for the full list of variables. (F) Nomogram for predicting 1‐, 3‐, and 5‐year OS for AML patients in TCGA cohort

The strong prognostic significance of *CD300A* status led us to hypothesize that it may add prognostic value to established risk models. Two gene expression‐based prognostic models‐LSC17 and LI24‐have shown their superior prognostic performance in risk stratification for AML patients.^39^, ^40^ We therefore test the predictive power of *CD300A* expression in the context of these two models. We established both models in the TCGA and GSE10358 cohorts and patients were stratified into high‐ and low‐risk groups, respectively (dichotomized according to the median score value). When applied to each risk group stratified by LSC17 and LI24 in the TCGA cohort, *CD300A* status was still able to discriminate between shorter and longer OS both within the high‐ and low‐risk groups (Figure 5A,B). This was also true for both models in the GSE10358 cohort (Figure S2A and B). We then compared the prediction performance of *CD300A* expression with that of LSC17 and LI24 using by calculating the AUC. The AUC of *CD300A* was 0.567, 0.640, and 0.710 in the TCGA cohort at 1, 3, and 5 years; *CD300A* yielded the highest AUC in predicting the 5‐year survival rate, even surpassing LSC17 and LI24 (Figure 5C). In the GSE10358 cohort, *CD300A* expression also had a good performance in predictive accuracy (AUC: 0.623, 0.681, and 0.696 at 1, 3, and 5 years, Figure S2C), comparable to that of LSC17 but lower than LI24. In summary, these results suggest *CD300A* as a good candidate for refining existing classification schemes.

### 3.6 Prognostic model of CD300A in AML

To assess whether *CD300A* expression impacted OS independent of known prognostic factors for AML, we performed multivariate analysis in the TCGA cohort. In multivariate models, including all parameters with p‐value less than 0.2 under univariate analysis (Figure 5D and Table S1), high expression of *CD300A* remained an independent prognosticator for OS (*p* = 0.031) together with age (*p* < 0.0001), white blood count (WBC count, *p* = 0.039), *TP53* mutation status (*p* = 0.001), *FLT3*‐ITD mutation status (*p* = 0.013), and cytogenetic risk group (*p* = 0.032) (Figure 5E).

To better predict AML patients' prognosis, we constructed a nomogram based on multivariable Cox proportional hazards model in the TCGA cohort (Figure 5F). Six independent prognostic factors, age, WBC, mutations of *TP53* and *FLT3*‐ITD, cytogenetic risk, and *CD300A* expression, were included in the model. In the nomogram, each variable is assigned a separate score, and the sum of these scores was rescaled to a range of 0–100 to estimate the probability of an event. The probability of AML patient survival at 1‐, 3‐, and 5‐year could be determined by drawing a line from the total point axis straight down to the outcome axis. As can be seen in the figure, the nomogram quantitatively predicted the probability of 1‐year, 2‐year, and 3‐year OS (Figures 5F).

### 3.7 Molecular subtypes and clinical characteristics associated with CD300s expression

**FIGURE 6:**
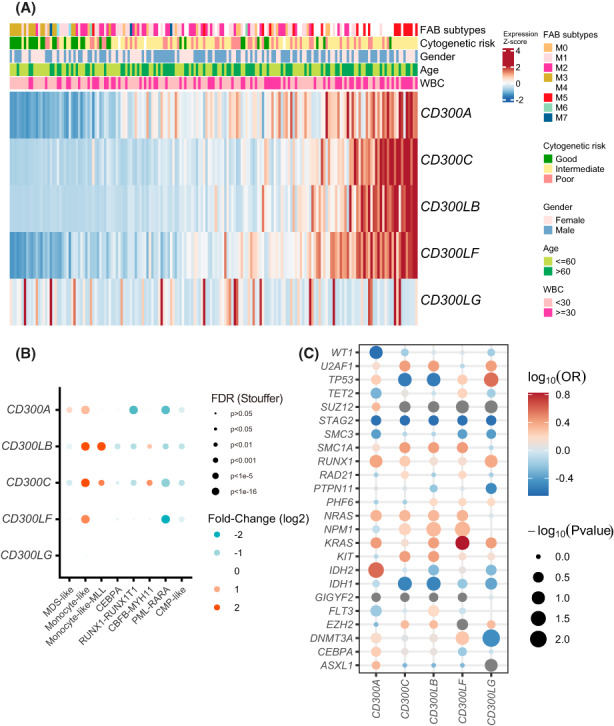
Molecular subtypes and clinical characteristics associated with *CD300s* expression. (A) Heatmap showing association between *CD300s* expression and clinical characteristics in the TCGA AML cohort. (B) Expression differences of *CD300s* among molecular subtypes in the Hemap AML dataset. The expression fold change between each subtype and the remaining samples were compared using the Wilcoxon rank sum test. The color of the dots indicates fold changes (log2) and size indicates the FDR values. The FDR values were categorized into six groups based on significance cutoffs for visualization (0.05, 0.01, 0.001, 1e‐5, 1e‐16). (C) Bubble plot showing associations between the expression of *CD300s* and common mutational events in the TCGA dataset. Bubble size indicates −log10 (Fisher test p‐value). Color signifies log10 (odds ratio [OR]), positive association is indicated with red circles, negative with blue circles, and non‐association with gray circles

We then examined the associations of *CD300s* expression with the clinical and genetic characteristics in the TCGA AML cohort. Significant differences were found between FAB subtypes and *CD300A*‐*CD300LF* expression status: high expression of *CD300A*‐*CD300LF* were significantly more frequent in AML with myelomonocytic (M4) and monocytic (M5) morphology, whereas the M3 subtype was exclusively observed in patients with low *CD300A*‐*CD300LF* expression (Figure 6A). Consistently, when examining the expression differences of *CD300s* across published molecular subtypes in the Hemap AML dataset, we found that *CD300A*‐*CD300LF* were more highly expressed in monocyte‐like AML, with low‐expressions levels in the PML‐RARA subtype (Figure 6B). Moreover, patients with high *CD300A*‐*CD300LF* expression were more likely to be >60‐year‐old and less likely to present with favorable cytogenetics (Figure 6A).

To determine whether *CD300s* correlated with mutational status of AML‐associated genes, we examined significantly mutated genes occurred in patients with high and low *CD300s* expression (dichotomized at the median expression value of each gene), using mutational data adopted from TCGA. As shown in Figure 6C, patients with high *CD300A* expression had higher frequency of mutations in *IDH2*, while *IDH1* was more frequently mutated in those with low *CD300LB* and *CD300C* expression. Patients with *NPM1* mutations had a significantly higher expression of *CD300LB* and *CD300LF* than those with *NPM1*‐wild type. Moreover, high *CD300LG* expression was positively correlated with *TP53* mutations and negatively correlated with *DNMT3A* mutations.

### 3.8 Association between CD300s expression and immune responses in cancer

**FIGURE 7:**
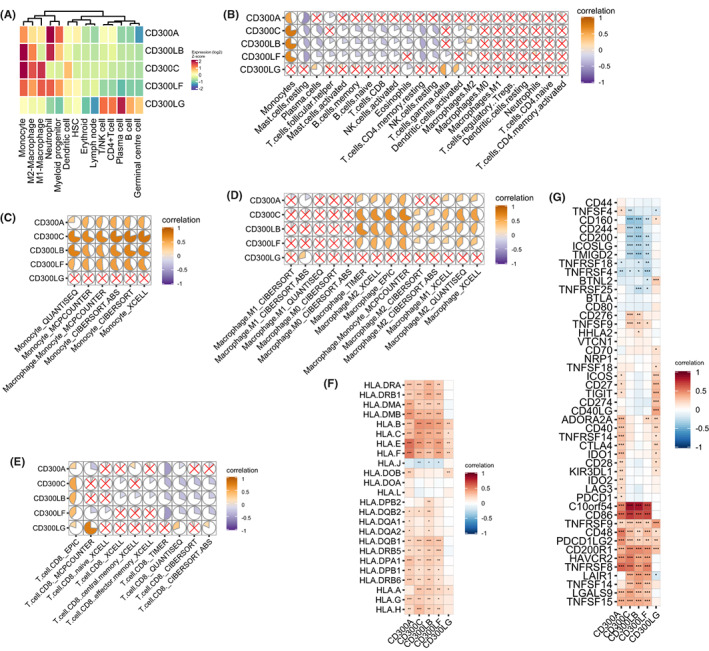
The relation between *CD300s* expression with immune cell infiltration, HLA genes, and immune checkpoints. (A) Heatmap showing *CD300s* expression in normal cell populations from the Hemap dataset. (B) Correlation matrix plot showing correlations between *CD300s* and tumor immune infiltrating cells in AML. The overall immune cell compositions were estimated by CIBERSORT in the TCGA dataset. (C‐E) Correlation matrix plots showing correlations between *CD300s* with monocytes (C), macrophages (D), and CD8 T cells (E). The overall immune cell compositions were estimated by indicated methods in the TCGA dataset. (F and G) Heatmap showing correlation between the expression of *CD300s* with HLA genes (F) and immune checkpoint genes (G) in the TCGA dataset

The *CD300* molecules are known to play important roles in the fine tuning of immune responses, we thus further explored the relationship between *CD300s* and tumor immune infiltrates. First, analysis of normal cell populations from the Hemap dataset revealed that *CD300s* were generally expressed at higher levels in myeloid lineage immune cells (monocytes, macrophages, neutrophils, and myeloid progenitors), with relatively low expression in lymphoid lineages (lymph node, T/NK cells, CD4+ T cells, plasma cell, B cell, and germinal center cell) (Figure 7A). Interestingly, the opposite trend was observed for *CD300LG*. This myeloid preference was also confirmed in two recently published scRNA‐seq datasets of AML (Van Galen AML scRNA and FIMM AML scRNA, Figures S3A and B). Moreover, we have observed a strong protein expression of *CD300A* and *CD300LF* in monocytes using Human Proteome Map (https://www.humanproteomemap.org/), a database for mass‐spectrometry proteomic analysis (Figure S4). It is noteworthy that *CD300A* showed a strong signal on NK cells in both the scRNA‐seq and mass‐spectrometry datasets (Figure S3 and S4), consistent with previous findings that *CD300A* is expressed on the surface of all human NK cells.^17^, ^18^

We then assessed the correlation of *CD300s* expression with infiltrations of 22 immune cell types inferred by the CIBERSORT algorithm. We found that *CD300A*‐*CD300LF* expression was positively associated with monocyte infiltrations but negatively associated with infiltrations of resting mast cells. *CD300LB*, *CD300C*, and *CD300LF* expression also showed significantly negative associations with infiltration of T/NK cells (CD8 T cells, resting T cells CD4 memory cell, activated, and resting NK cells) and B cells (plasma cells, memory B cells, and naïve B cells), while these three genes showed a significantly positive association with the immunosuppressive M2 macrophages (Figure 7B). By contrast, no correlation was found of *CD300A* and *CD300LG* expression with the majority of cell types (Figure 7B). Similar results were found by analyzing the CIBERSORT estimates in the GSE10358 and GSE6891 dataset (Figure S5A and B). Importantly, when other methods were used for calculating the relative fractions of immune cells, positive associations between *CD300A*‐*CD300LF* and monocytes were consistently seen, while M2 macrophages were positively correlated and CD8 T cells negatively correlated with *CD300A*‐*CD300LF* for most‐if not all‐instances in all three datasets (Figure 7C‐E and Figure S6A‐F). Collectively, these findings indicate immunosuppressive roles of *CD300s* in the TME.

### 3.9 Correlation of CD300s with HLA genes and immune checkpoints in AML

Given that human leukocyte antigen (*HLA*) and immune checkpoints plays important roles in the TME, it is therefore of great interest to evaluate the relationship between *CD300s* and these immune signatures. We found that *CD300A*‐*CD300LF* positively correlated with most of the *HLA* genes (Figure 7F). Since cancers with higher *HLA* gene expression were often more immunologically active, showing significantly higher immune cell infiltration,^41^ it is reasonably to speculate that high *CD300s* expressers might share the same trait. In addition, we observed strong positive correlations between *CD300A*‐*CD300LF* expression and immune checkpoint molecules such as: *C10orf54*, *CD86*, *CD200R1*, *HAVCR2*, *TNFRSF8*, and *TNFRSF9*; whereas *CD300C*, *CD300LB*, and *CD300LF* expression were negatively correlated with *CD160*, *CD200*, *ICOSLG*, and *TMIGD2* (Figure 7G). Distinct correlation patterns were observed between *CD300LG* and these signatures, a trend consistent with the previous results (Figure 7F,G).

### 3.10 CD300s expression predicted response to immunotherapies

**FIGURE 8:**
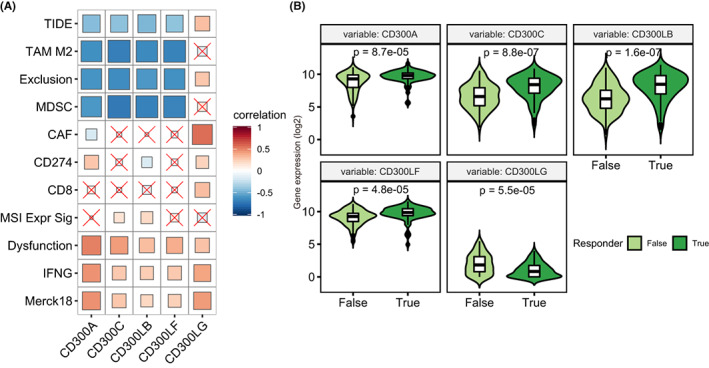
*CD300s* expression predicted response to immunotherapies. (A) Correlation matrix showing the association between *CD300s* expression with T‐cell dysfunction and T‐cell exclusion signatures in TCGA AML cohort, as determined using the tumor immune dysfunction and evasion (TIDE) method. (B) Violin plots comparing the expression of *CD300s* between patients who benefitted or did not benefit from immunotherapy in AML, as predicted by the TIDE algorithm. Significances were calculated by Wilcoxon rank sum tests

Recently, a gene expression‐based score called tumor immune dysfunction and exclusion (TIDE) were showed to have excellent performance in predicting immunotherapy clinical response.^42^ We therefore checked the relationship of *CD300s* with the expression signatures of T‐cell dysfunction and T‐cell exclusion. Surprisingly, we found a negative correlation of *CD300A*‐CD330LF with T cell exclusion signatures, including myeloid‐derived suppressor cells (MDSCs), M2 subtype of tumor‐associated macrophages (TAMs), exclusion and TIDE score but a positive correlation with the T‐cell dysfunction score, IFNG, and merck18 signatures (Figure 8A). This indicates that *CD300* molecules might contribute to immune evasion through the induction of T‐cell dysfunction. Based on these results, we asked whether *CD300* molecules would have a guiding value in predicting immunotherapy in AML. In the TCGA AML cohort, we found that the predicted responders showed significantly higher expression levels of *CD300A*‐*CD300LF* but lower levels of *CD300LG* than non‐responders (Figure 8B), suggesting *CD300* expression could be a good predictor for immunotherapy in AML.

### 3.11 The biological significance of CD300s expression in AML

**FIGURE 9:**
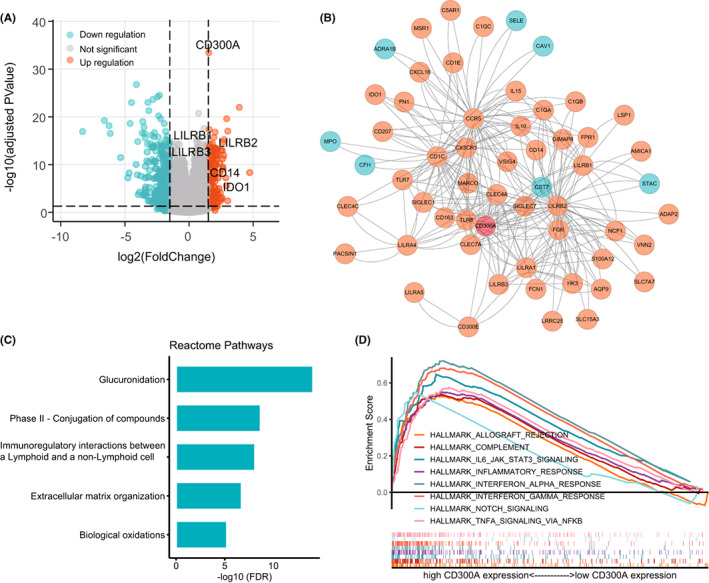
The biological significance of *CD300A* expression in AML. (A) Volcano plot showing differentially expressed genes (DEGs) between high and low *CD300A* expressers. (B) Cytoscape analysis of *CD300A*‐related network using PPI information obtained from STRING database (http://stringdb.org/). Red nodes represent upregulated genes and blue represent downregulated genes. (C) Reactome Pathway analysis of DEGs. (D) Gene set enrichment analysis (GSEA) of patients with high and low *CD300A* expression, using Hallmark gene sets obtained from the Molecular Signatures Database (MSigDB)

Next, we investigated the potential biological features associated with *CD300s* in AML. We choose *CD300A* to represent this family, since the expression of five *CD300* members (except for *CD300LG*) were highly correlated in AML (Figure S7). A comparison of gene expression profiles of patients with high and low *CD300A* expression (based on the median expression value) was then performed. Overall, 442 genes (147 up‐ and 295 downregulated; adjusted *p* < 0.05; |log2FC| > 1.5) were differentially expressed in *CD300A* ^high^ versus *CD300A* ^low^ patients (Figure 9A and Data S1). Among the genes positively correlated with *CD300A* were monocytes marker gene *CD14*, further supporting the monocytic prevalence of *CD300s*. Also highly correlated were well‐characterized immune checkpoint genes in AML, such as *IDO1*, *LILRB1*, *LILRB2*, and *LILRB3* (Figure 9A). Using the STRING tool, we constructed a protein–protein interaction (PPI) network of the differentially expressed genes (DEGs), with a confidence score >0.90. Genes interacted with *CD300A* and their sub‐networks were shown through Cytoscape software (Figure 9B). We observed that 13 genes were directly interacting with *CD300A*: *LILRB1*, *LILRB2*, *LILRB3*, *LILRA1*, *LILRA4*, *CLEC4A*, *CCR5*, *SIGLEC7*, *CX3CR1*, *CD1C*, *CST7*, *CD300E*, and *FGR*. The majority of these genes were involved in immune responses. Among them were immune‐inhibitory leukocyte immunoglobulin‐like receptors: *LILRB1*, *LILRB2*, *LILRB3*, *LILRA1*, *LILRA4*;^43^ *CX3CR1*, and *CD1C*, commonly expressed in myeloid dendritic cells (mDCs), which modulate T‐cell functions with either stimulatory or suppressive effects;^44^, ^45^ *SIGLEC7*, an inhibitory receptor expressed on NK cells that could indicate NK cell dysfunction in AML.^46^, ^47^ Genes involved in the pathogenesis of AML such as *CCR5*,^48^ *CST7*,^49^ and *FGR* ^50^ were also detected. Collectively, these gene–gene interactions might contribute to the immunomodulatory effects of *CD300A* in AML.

GeneMANIA analysis confirmed that *LILRB3* was closely connected with *CD300* molecules, and that the co‐expressed genes were mainly involved in negative regulation of a series of cellular processes such as: mononuclear cell proliferation, leukocyte proliferation, leukocyte activation, cell activation, immune system process, and lymphocyte activation, highlighting the immunosuppressive functions of *CD300* members (Figure S8A).

To further identify the potential function of these DEGs, we performed GO analysis using these DEGs and the top 10 significant terms of BP, MF, and CC enrichment analysis are shown (Figure S8B). Most GO terms were related to receptor activity, component of plasma membrane, and plasma membrane. KEGG analysis revealed that metabolism‐related pathways were significantly enriched (Figure S8C); while Reactome Pathway analysis demonstrated the enrichment of immunoregulatory interactions between a lymphoid and a non‐lymphoid cell (Figure 9C). Further, using GSEA, we found that multiple immune‐related cancer hallmarks were enriched in the *CD300A* ^high^ group, such as HALLMARK_ALLOGRAFT_REJECTION, HALLMARK_COMPLEMENT, HALLMARK_IL6_JAK_STAT3_SIGNALING, HALLMARK_INFLAMMATORY_RESPONSE, HALLMARK_INTERFERON_ALPHA_RESPONSE, HALLMARK_INTERFERON_GAMMA_RESPONSE, HALLMARK_NOTCH_SIGNALING, HALLMARK_TNFA_SIGNALING_VIA_NFKB (Figure 9D).

## 4 DISCUSSION

The *CD300* receptor family members are a group of genes involved mainly in fine‐tuning the immune responses. Specifically, *CD300A* and *CD300LF* have long cytoplasmic tail containing ITIMs that are capable of eliciting negative signals.^15^ While *CD300LF* could act both as activating and inhibitory receptor, *CD300A* preferentially exerts immune‐inhibitory activity. Since our analyses were largely based on microarray datasets, where the probes of *CD300LD* and *CD300E* does not exist, we therefore focused on investigating the other five members (*CD300A*, *CD300LB*, *CD300C*, *CD300LF*, and *CD300LG*). First, using GTEx database, we showed that *CD300A*‐*CD300LF* were predominantly expressed in the blood, lung, and spleen tissue, consistent with previous reports.^51^ One exception is *CD300LG*, which was highly expressed in the adipose tissue, breast, heart, and testis, but it was not expressed by blood. This is in line with the finding that *CD300LG* was not expressed by leukocytes but was expressed at high levels in the heart.^52^ Indeed, in all our following analyses, *CD300LG* showed a distinct transcriptional/clinical pattern compared with the other four genes. This could be due to the structural difference observed in *CD300LG*: it lacks structural motifs of stimulatory or inhibitory potential and contains a mucin‐like domain.^13^, ^51^

Several studies have reported the pathological roles of *CD300s* in autoimmune diseases,^21^, ^22^, ^23^, ^24^, ^25^ but there is still insufficient data for their involvement in human cancer development. To date, transcriptional dysregulations of *CD300s*, especially *CD300A* and *CD300LF*, have been reported mostly in patients with hematological malignancies, such as ALL, AML, and DLBCL.^26^, ^27^, ^28^, ^29^, ^53^, ^54^ Accordingly, our analyses showed that *CD300A*‐*CD300LF* were remarkably overexpressed in AML both in RNA‐seq (TCGA) and microarray (GSE63270) datasets. In addition, we found that *CD300A*‐*CD300LF* were more often upregulated in tumors, whereas the opposite was seen for *CD300LG*, further supporting the distinct expression pattern of *CD300LG*.

Transcriptional control of the *CD300* molecules is essential for the immediate regulation of immune responses. Various triggering stimuli, including cytokines,^21^, ^55^ Toll‐like receptors (TLRs),^56^ hypoxia, and granulocyte–macrophage‐colony‐stimulating factor (GM‐CSF),^57^ are known to regulate *CD300s* expressions. However, evidences regarding their transcriptional regulation at the genomic and epigenetic level is insufficient. We thus assessed the genomic and epigenetic landscape of *CD300s* across TCGA pan‐cancer studies. Interestingly, we could not identify any mutation or copy number changes of *CD300s* in AML. Further analyses revealed that *CD300A* and *CD300LF* were also unlikely to be regulated by DNA methylation in AML. Recent evidence indicates that *CD300A* could be robustly induced by PPARβ/δ‐mediated histone modification in macrophages.^38^ Similarly, we observed a significant enrichment of H3K4me3 marks in the promoter regions of *CD300A* gene in leukemia cell lines. These findings indicate that *CD300A* might be transactivated by H3K4me3 via direct binding.

Among *CD300* family members, *CD300A* was found to be negatively associated with prognosis in ALL,^27^ AML,^28^ and DLBCL.^29^ In the present study, we comprehensively assessed the prognostic implications of *CD300s* in 33 cancer types and we were able to confirm the adverse prognostic impact of *CD300A* in AML. In the previous study, survival analysis of *CD300A* was restricted to the TCGA dataset, our further used seven independent AML datasets and a meta dataset including 1115 AML patients to confirm the prognostic value of *CD300A*. Moreover, we demonstrated additional value of *CD300A* expression in refining existing classification schemes in AML. These results suggest *CD300A* as a promising biomarker of cancer risk in AML patients.

As immune‐modulatory molecules, it is of particular interest to investigate *CD300s*' correlations with immune cell infiltrations in the TME. *CD300A*‐*CD300LF* were reported to be preferentially expressed in cells belonging to the myeloid lineage.^13^, ^15^, ^16^ In accordance, we showed that *CD300A*‐*CD300LF* were highly expressed in myeloid lineage immune cells (especially monocytes) both in bulk or at the single cell level. Besides, *CD300A*‐*CD300LF* were significantly enriched in monocyte‐like AML subsets. It was noteworthy that *CD300A* was also highly expressed in NK cells infiltrating AML cells, a finding similar to what has been reported previously.^17^, ^18^ It has been known that patients with AML often have dysfunctional T cells and NK cells at diagnosis.^58^, ^59^ Indeed, *CD300A* could inhibit NK cell‐mediated cytotoxicity and make NK cells display a similar status with exhausted T cells.^17^, ^18^ This facilitates AML blasts escape from immune elimination and cooperate to promote disease progression, which might explain the poor outcome observed in high *CD300A* expressers. We can safely speculate that blocking *CD300A* could restore NK cells activity and lead to AML suppression. Accordingly, we found *CD300A* expression was positively correlated with T‐cell dysfunction score and high *CD300A* expressers were predicted to have a better response to immunotherapy in AML. Also, *CD300A* was found to be positively associated with inhibitory immune checkpoints, such as *C10orf54* (also known as *VISTA*), *CD86*, *CD200R1*, *HAVCR2* (also known as *Tim‐3*), and the *LILRB* family genes (please refer to our unpublished work, DOI: 10.21203/rs.3.rs‐810,313/v1), all of which were reported to be overexpressed in AML cells and some of them may become promising therapeutic targets.^60^, ^61^, ^62^, ^63^, ^64^, ^65^ Interestingly, two genes co‐expressed and interacted with *CD300A*‐*CX3CR1* and *CD1C*‐were both found to be expressed on mDCs in the AML TME. While *CX3CR1*+ DC (like *CD206*+ DC) expresses high levels of *CD274* (*PD‐L1*) and *PDCD1LG2* (*PD‐L2*) and mediates T‐cell suppression, the *CD1C*+ DC population expresses mainly functional molecules, and displays a T‐cell stimulatory capacity.^44^ This further illustrates the fine‐tuning of immune responses in AML by *CD300A*‐related gene networks.

Our study has several limitations. First, because the expression data of *CD300LD* and *CD300E* were not obtainable from microarray data sets, their clinical and immunological implications were leaved undetermined. Second, *CD300A* has already been reported to be upregulated and prognostically significant in AML.^28^ Nonetheless, our results are important in providing a comprehensive view of the prognostic value of *CD300* members across a broad type of cancers. Moreover, we have extended the previous finding by validating the prognostic impacts of *CD300A* in seven independent AML cohorts and comparing its predictive performance with established models. Third, most of our findings were based on RNA‐seq and microarray datasets, future validation using techniques like qPCR and immunohistochemistry (IHC) is needed. Finally, although our bioinformatics analyses gave some immunological insights of *CD300s* in AML and demonstrated their potential as predictive biomarkers for immunotherapy, experiment‐based information, and prospective clinical assessment are clearly required.

In summary, we found that *CD300A*‐*CD300LF* were significantly upregulated and high expression of these genes predicted worse survival in AML. Specifically, *CD300A* may aid risk stratification in AML and its expression was likely to be regulated by H3K4me3 histone modification. Importantly, we demonstrated *CD300A* as an essential co‐inhibitory signal that might cause NK cell exhaustion and promotes immune escape of AML cells. *CD300A* may be a potential candidate for AML therapy via monoclonal antibodies or other specific inhibitory strategies. Prospective clinical study is clearly needed to validate the findings in our study.

## AUTHOR CONTRIBUTIONS

JQ and JL conceived and designed the study; Z‐JX, YJ, X‐LZ, and P‐HX collected and assembled data; Z‐JX, X‐MW, and J‐CM performed data analysis; Z‐JX drafted the manuscript; JQ and JL participated in study supervision and commented on the manuscript. All authors read and approved the final manuscript.

## CONFLICT OF INTEREST

The authors declare that they have no competing interests.

## Supporting information


Data S1

Supinfo S1

## Data Availability

The datasets analyzed in this study are available in the following open access repositories: GTEx, www.gtexportal.org/ CCLE, https://www.broadinstitute.org/ccle TCGA, https://portal.gdc.cancer.gov/, http://www.cbioportal.org UCSC Xena, https://xena.ucsc.edu/ UALCAN, http://UALCAN.path.uab.edu/ GEO, https://www.ncbi.nlm.nih.gov/geo/ (GEO accession numbers: GSE63270, GSE116256, GSE6891, GSE10358, GSE37642, GSE12417, GSE71014, GSE74359, GSE89336, and GSE109619). FIMM AML scRNA data, https://www.synapse.org (doi: 10.7303/syn21991014). Human Proteome Map, https://www.humanproteomemap.org/ DiseaseMeth, http://bio‐bigdata.hrbmu.edu.cn/diseasemeth/analyze.html TIMER 2.0, http://timer.comp‐genomics.org/ GSCALite, http://bioinfo.life.hust.edu.cn/web/GSCALite/
