# Supplementary material for: Pan‐cancer analysis identifies CD300 molecules as potential immune regulators and promising therapeutic targets in acute myeloid leukemia
Source: Cancer Med. 2022 May 31;12(1):789–807. doi: 10.1002/cam4.4905 (PMC9844665; doi:10.1002/cam4.4905)
Supplement: Supplementary file 2 — Supinfo S1 [file CAM4-12-789-s002.docx]

**Supplementary Information**

**Pan-cancer analysis identifies *CD300* molecules as potential immune regulators and promising therapeutic targets in acute myeloid leukemia**

**This file contains Supplementary Figures (Supplementary Figures S1-S8) and Supplementary Tables (Tables S1).**


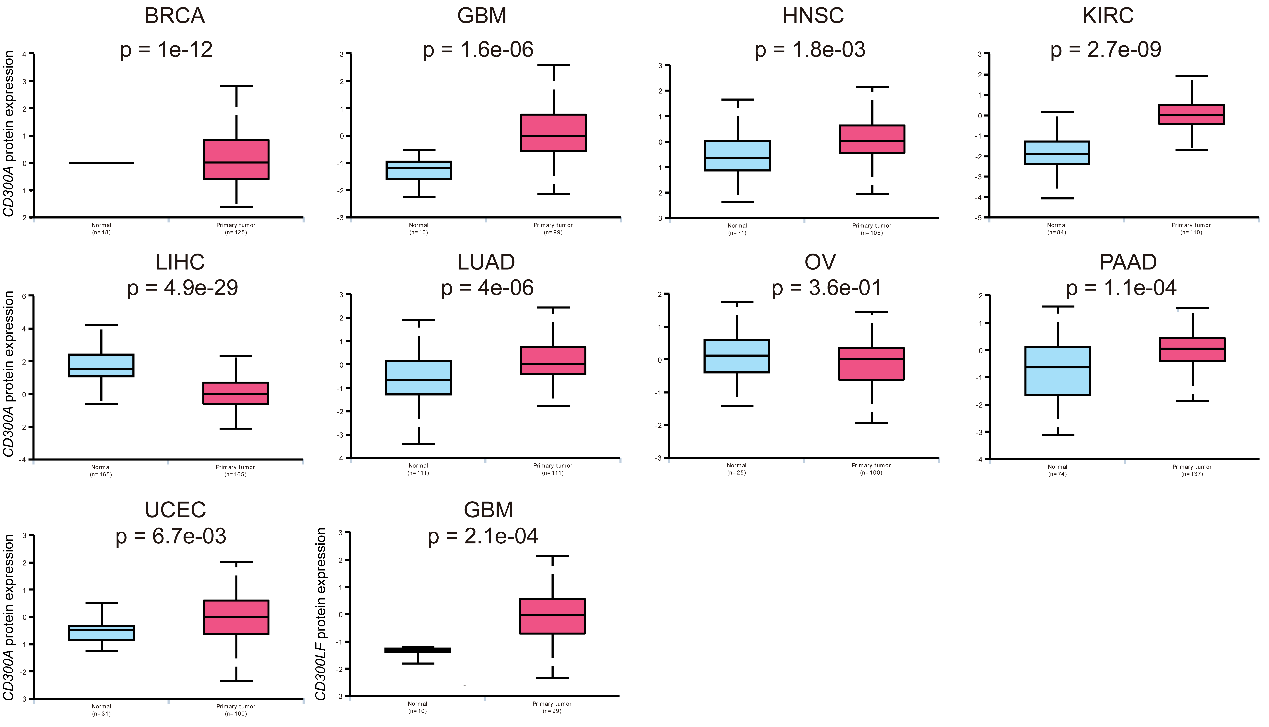


**Supplementary Figure S1. Boxplots showing protein expression differences of *CD300A* and *CD300LF* between indicated tumor and normal tissue samples, using proteomic dataset from the UALCAN (http://UALCAN.path.uab.edu/) database.** Blue, normal control samples; red, tumor samples.


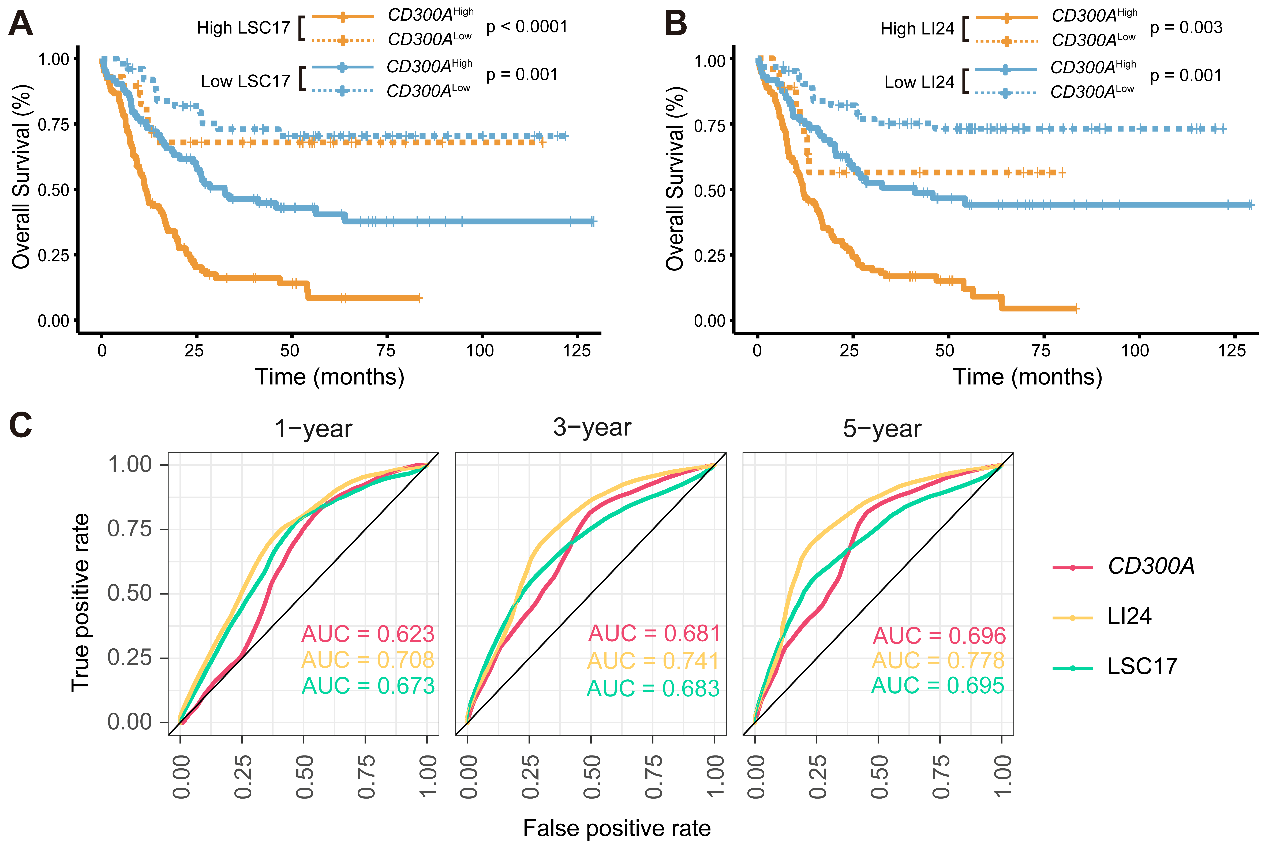


**Supplementary Figure S2. Additional value of *CD300A* expression in refining risk stratification in AML.** **(A and B)** OS of patients from GSE10358 as stratified by the LSC17 **(A)** and the LI24 **(B)** signature. Patients with a low and high risk score were further dichotomized according to *CD300A* expression status. **(C)** Time dependent ROC curves of *CD300A* expression, LSC17, and LI24 in the GSE10358 cohort at 1, 3 and 5 years.


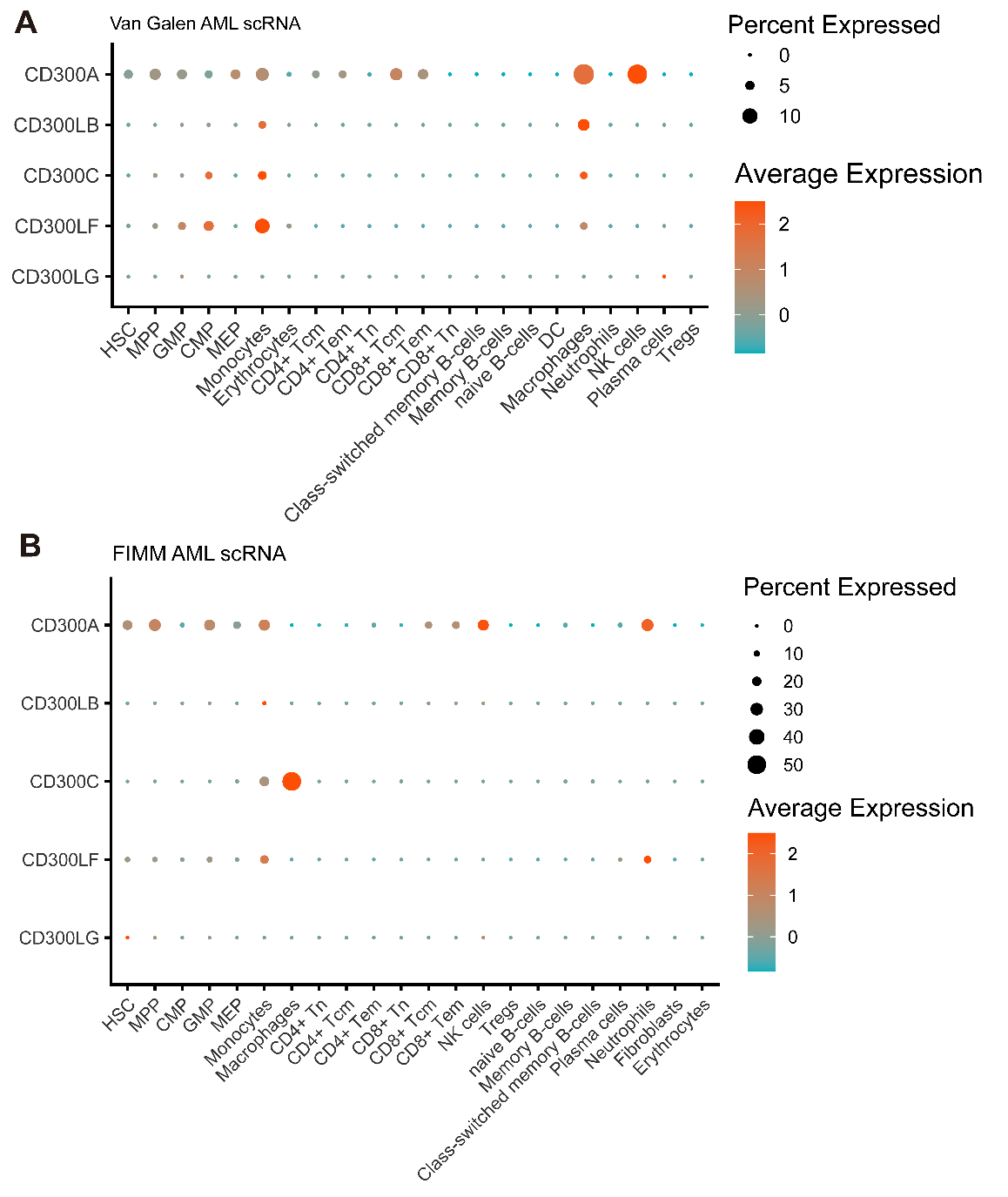


**Supplementary Figure S3. The expression patterns of *CD300s* in immune cells at the single cell level.** Dot plot showing expression pattens of *CD300s* in annotated cell types from Van Galen AML scRNA dataset **(A)** and FIMM AML scRNA dataset **(B)**. The color of the dots indicates average expression and size indicates percentage of cells with detectable expression.


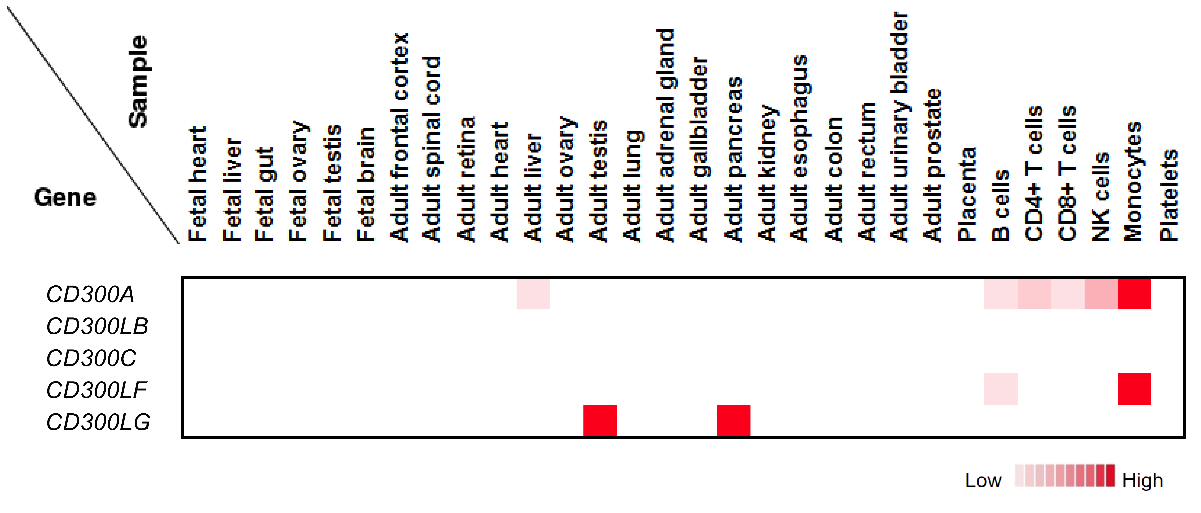


**Supplementary Figure S4.** **Protein expression levels of *CD300s* in normal organs/tissues and cell types as assessed from the Human Proteome Map (https://www.humanproteomemap.org/) database.** These include 17 adult tissues, 6 primary hematopoietic cells and 7 fetal tissues. Expression levels of *CD300s* are depicted by a heat map and red indicates higher expression.


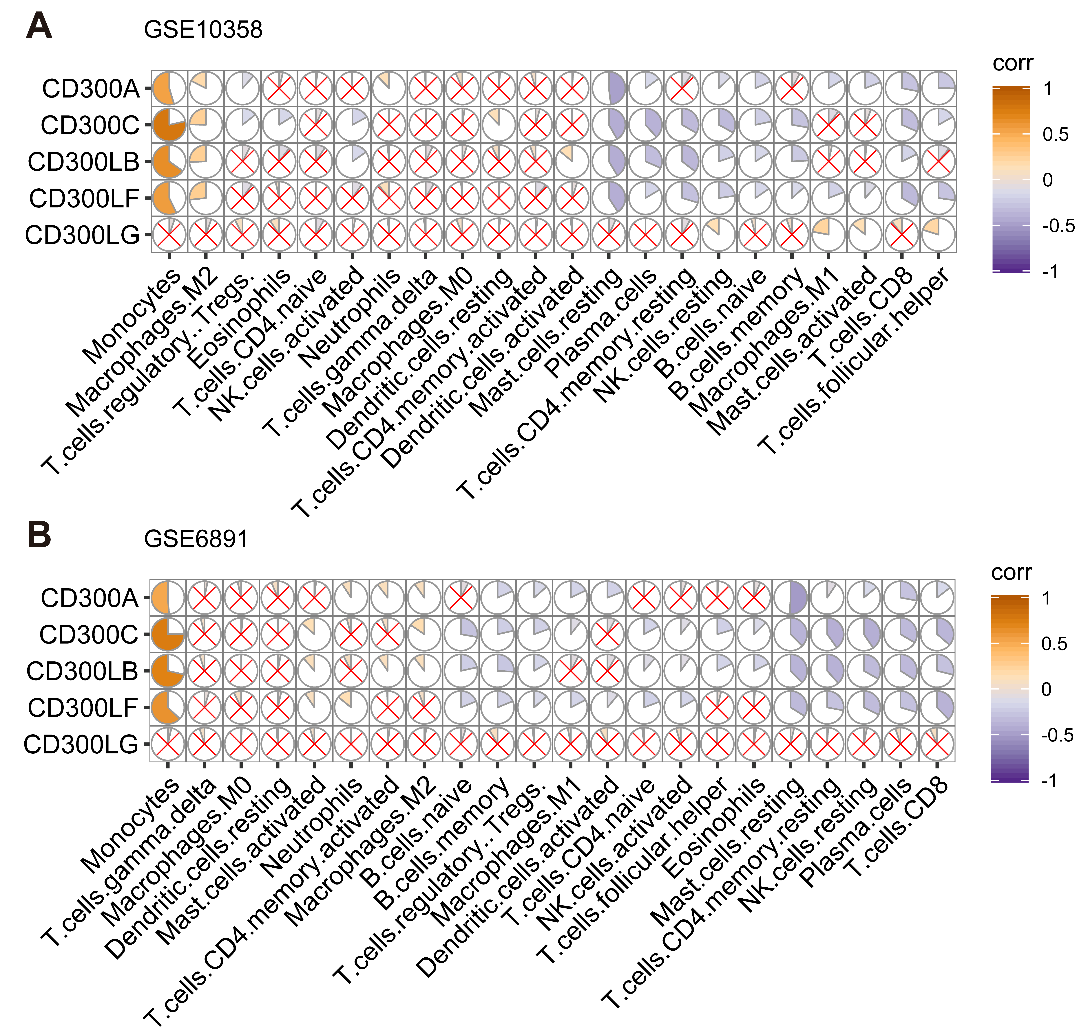


**Supplementary Figure S5. Correlation matrix plot showing correlations between *CD300s* and tumor immune infiltrating cells in AML.** The overall immune cell compositions were estimated by CIBERSORT in the GSE10358 **(A)** and GSE6891 **(B)** datasets.
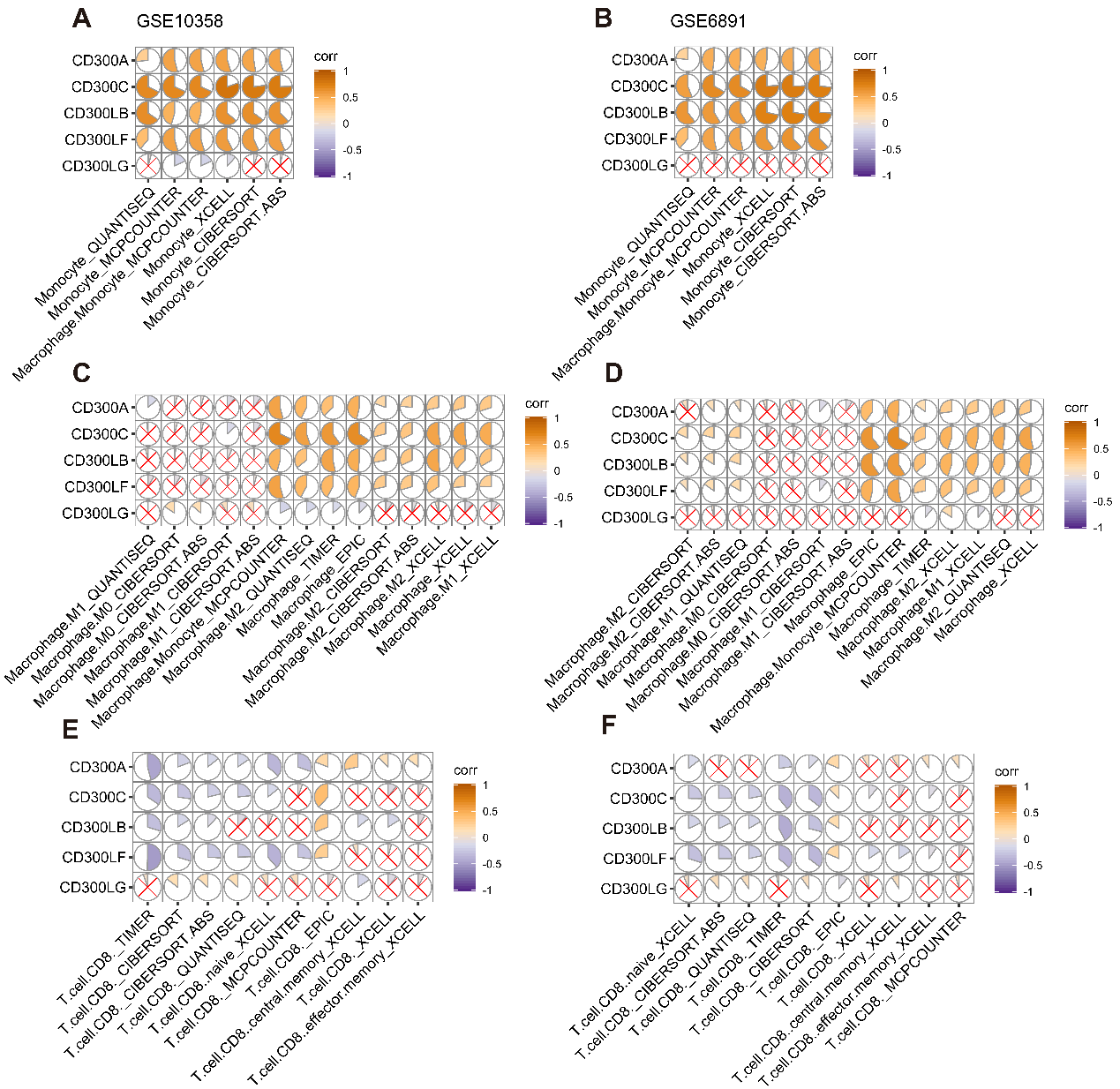


**Supplementary Figure S6. The relation between *CD300s* expression with immune cell infiltration. (A-F)** Correlation matrix plots showing correlations between *CD300s* with monocytes **(A and B)**, macrophages **(C and D)**, and CD8 T cells **(E and F)**. The overall immune cell compositions were estimated by indicated methods in the GSE10358 (left panel) and GSE6891 (right panel) datasets.


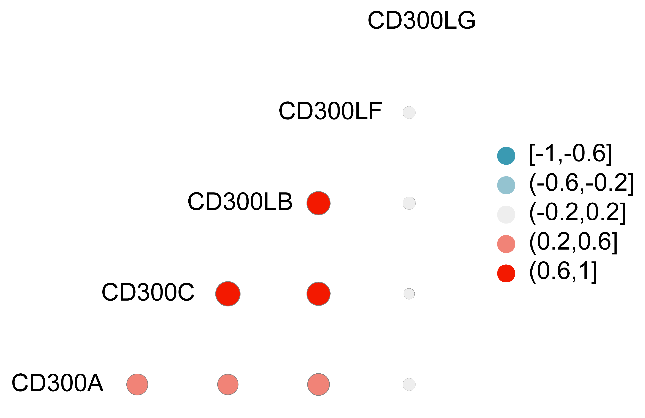


**Supplementary Figure S7. Correlation matrix of the expression of *CD300A*-*CD300LG* in the TCGA AML dataset.**


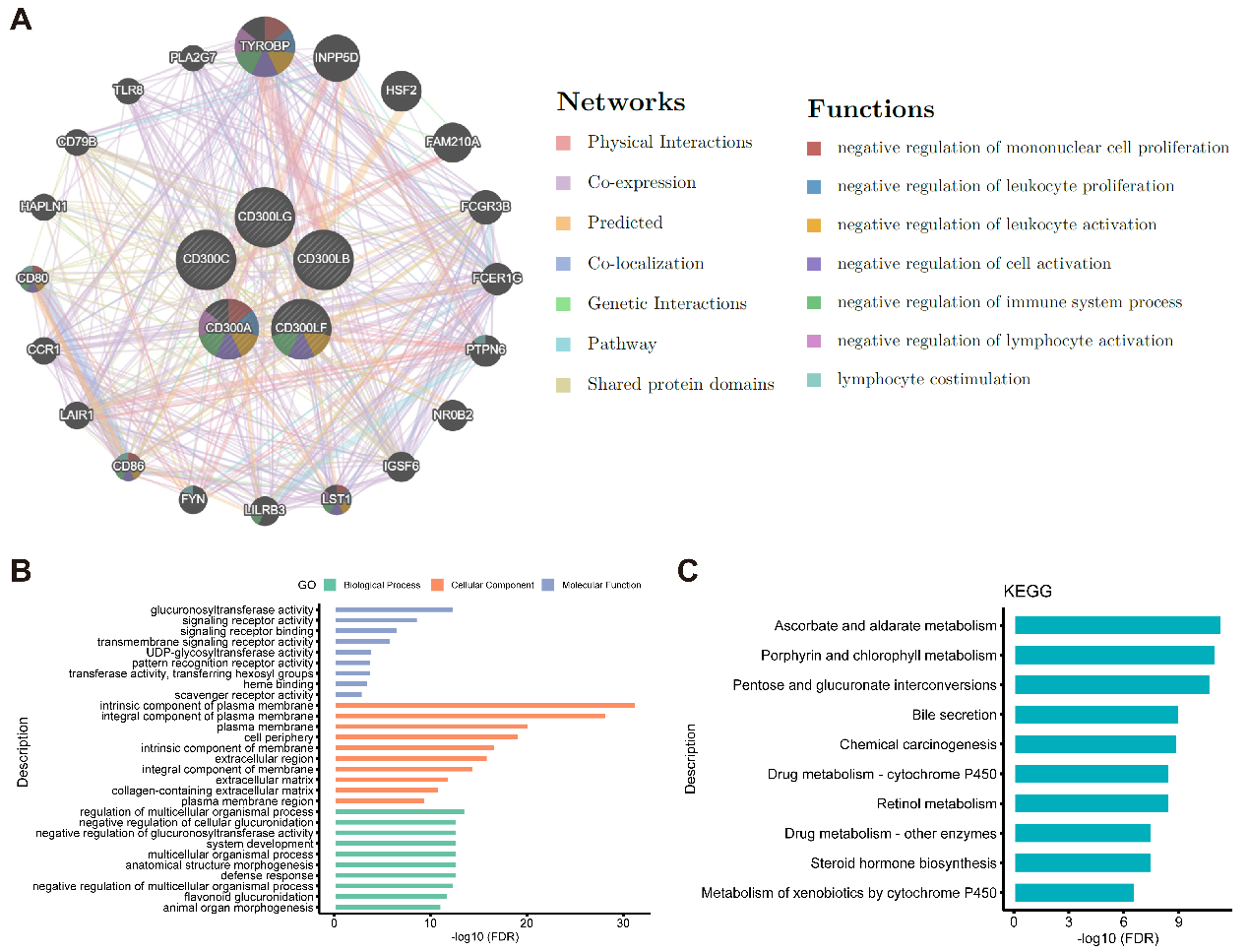


**Supplementary Figure S8. The biological significance of *CD300s* expression in AML. (A)** The gene network associated with the *CD300* gene family visualized through GeneMANIA. The colored patches on the circle indicate the function of the gene. **(B and C)** GO **(B)** and KEGG **(C)** analysis of DEGs.

# Supplementary Tables

# Table S1. Univariate analysis of *CD300A* expression for overall survival in the TCGA cohort

| **Variables^a^** | **Overall survival** | |
| --- | --- | --- |
|  | **Hazard Ratio (95% CI)** | ***P*** |
| **TCGA cohort** | **(n = 173)** | |
| *CD300A*^b^ | 3.434 (1.918-6.150) | **< 0.0001** |
| Age | 1.041 (1.026-1.056) | **< 0.0001** |
| WBC count | 1.004 (1.000-1.008) | **0.031** |
| Cytogenetic risk^c^ | 2.023 (1.496-2.736) | **< 0.0001** |
| *FLT3*-ITD^d^  *DNMT3A*^e^ | 1.361 (0.907-2.042)  1.596 (1.058-2.407) | **0.137**  **0.026** |
| *NPM1*^e^ | 1.182 (0.787-1.777) | 0.420 |
| *IDH2*^e^  *IDH1*^e^  *TET2*^e^  *RUNX1*^e^  *TP53*^e^  *NRAS*^e^ | 1.050 (0.575-1.916)  0.722 (0.365-1.428)  1.012 (0.529-1.939)  1.636 (0.913-2.932)  4.513 (2.469-8.250)  0.805 (0.374-1.730) | 0.875  0.349  0.971  **0.098**  **< 0.0001**  0.578 |
| *CEBPA*^e^ | 0.944 (0.477-1.866) | 0.868 |

Abbreviations: TCGA, The Cancer Genome Atlas; CI, confidence interval; WBC, white blood cells; ITD, internal tandem duplication.

NOTE: Hazard Ratio > 1 or Hazard Ratio < 1 indicate a higher or lower risk. p ≤ 0.20 were highlighted.

^a^Variables include clinical parameters (age, WBC count, and cytogenetic risk group) and ten 10 most frequently mutated genes in TCGA AML^1^.

^b^High vs low expression.

^c^Adverse vs intermediate vs favorable.

^d^Present vs absent.

^e^Mutated vs wild type.

**References**

1 Wen, X. M. *et al.* Association Analyses of TP53 Mutation With Prognosis, Tumor Mutational Burden, and Immunological Features in Acute Myeloid Leukemia. *Frontiers in immunology* **12**, 717527, doi:10.3389/fimmu.2021.717527 (2021).
